# Supplementary material for: Crosstalk between BER and NHEJ in XRCC4-Deficient Cells Depending on hTERT Overexpression
Source: Int J Mol Sci. 2024 Sep 27;25(19):10405. doi: 10.3390/ijms251910405 (PMC11476898; doi:10.3390/ijms251910405)
Supplement: Supplementary file 1 [file ijms-25-10405-s001.zip › ijms-3190407-supplementary.pdf]

TIG-1

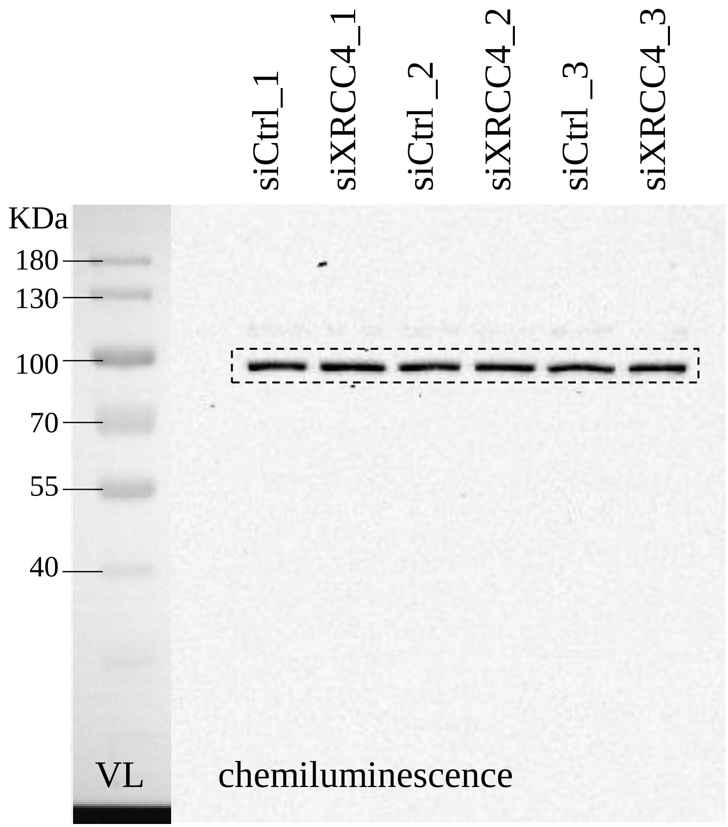

1° anti-**Ku80** 1:1000

2° anti-mouse 1:10000

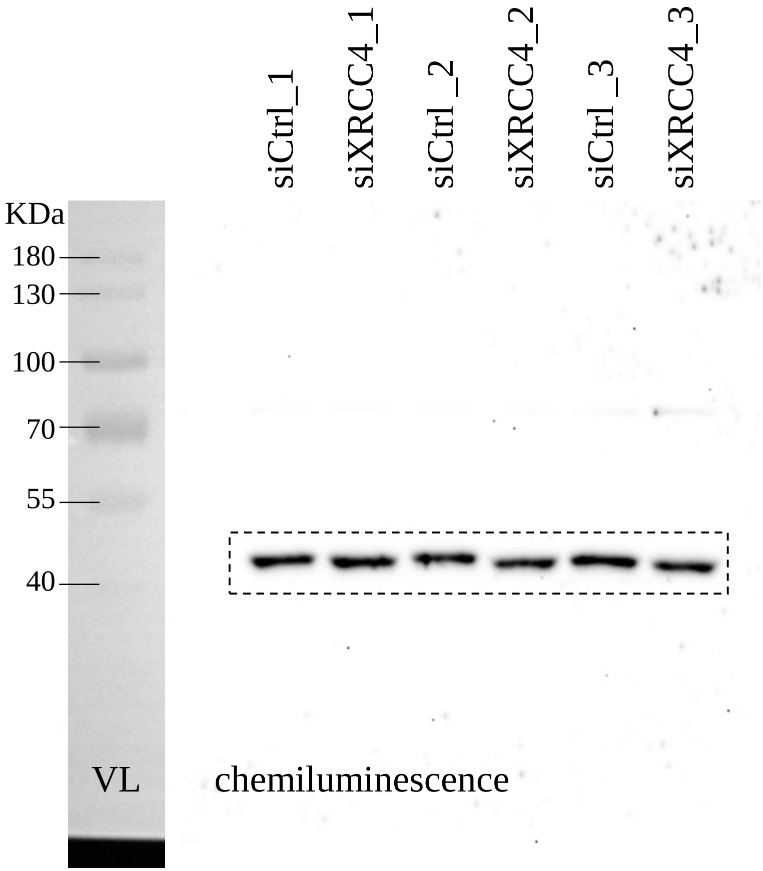

1° anti-**β-actin** 1:10000

2° anti-mouse 1:10000

Part of the image used for the densitometry in the Figure 1.

protein concentration (ug/ul)
siCtrl_1: 5.33, siCtrl _2: 6.19, siCtrl _3: 6.34;
siXRCC4_1: 5.40, siXRCC4_2: 5.93 siXRCC4_3: 6.85.
25ug of cell total protein lysate/lane

Figure S1: Original Western blot images corresponding to Figure 1.

TIG-1

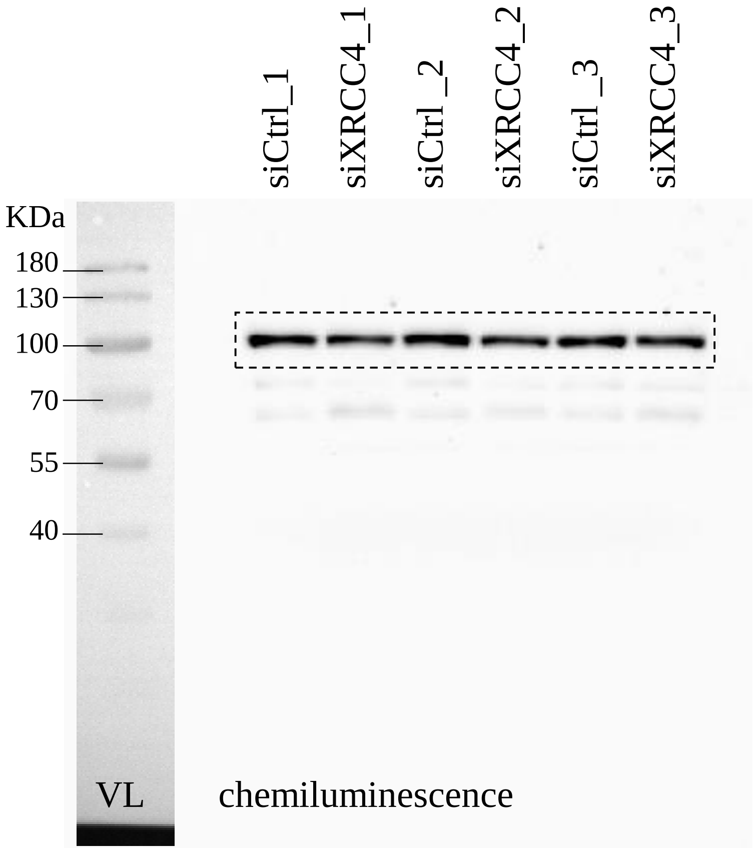

1° anti-**XRCC1** 1:2500

2° anti-mouse 1:10000

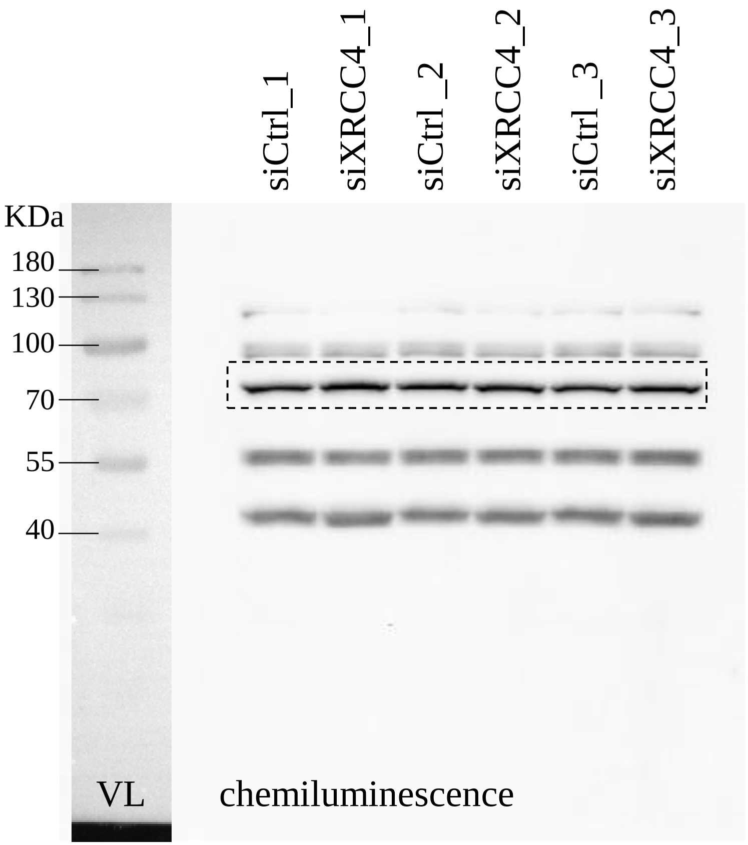

1° anti-**Ku70** 1:1000

2° anti-mouse 1:10000

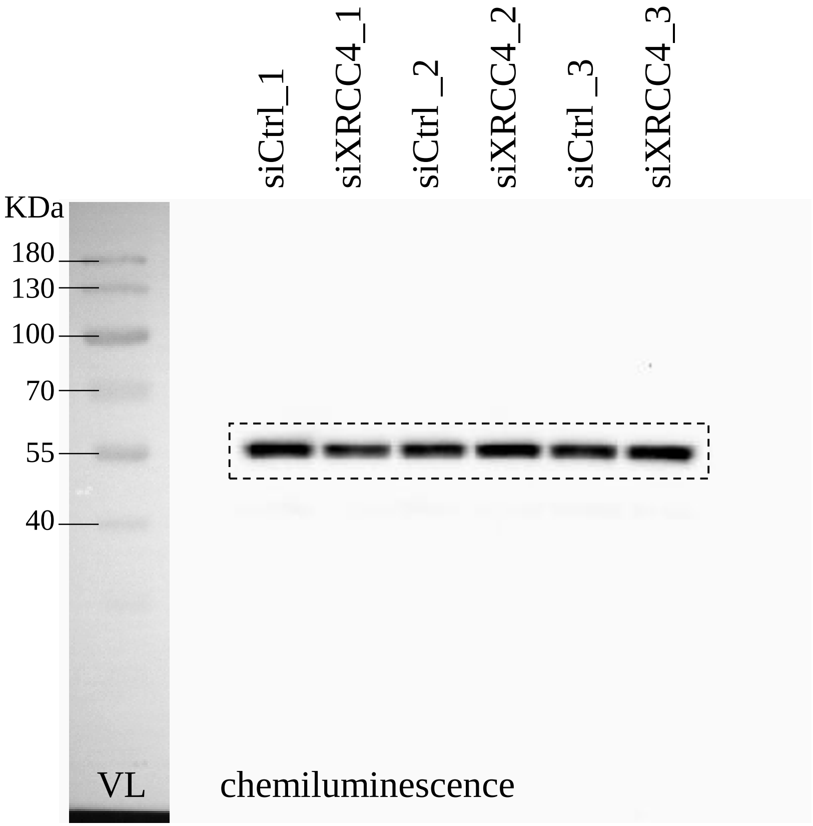

1° anti-**α-tubulin** 1:5000

2° anti-mouse 1:10000

[dashed box] Part of the image used for the densitometry in the Figure 1.

Figure S2: Original Western blot images corresponding to Figure 1.

protein concentration (ug/ul)
siCtrl_1: 5.33, siCtrl _2: 6.19, siCtrl _3: 6.34;
siXRCC4_1: 5.40, siXRCC4_2: 5.93 siXRCC4_3: 6.85.
25ug of cell total protein lysate/lane

TIG-1

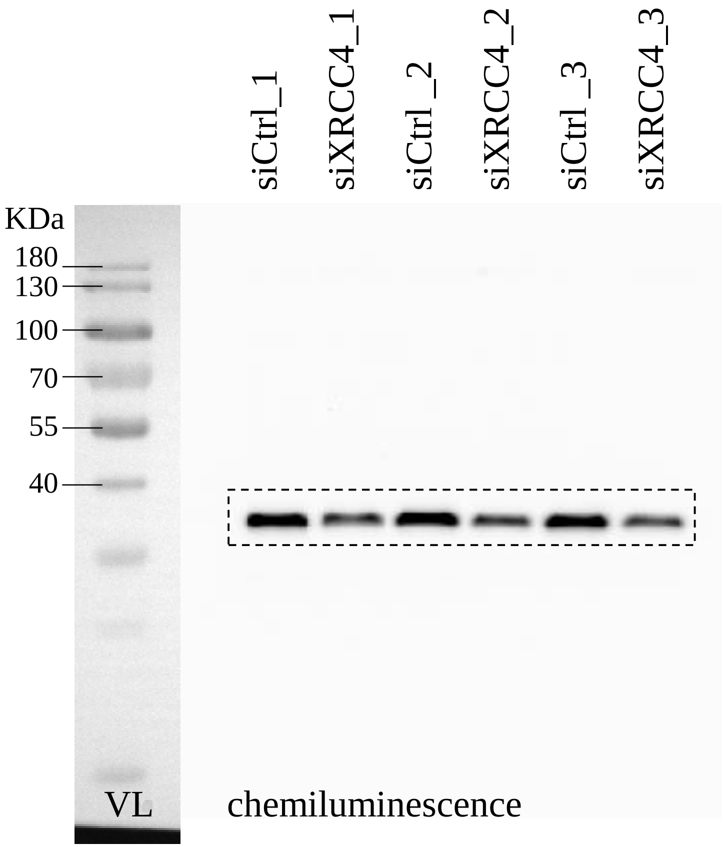

1° anti-**APE1** 1:2000

2° anti-rabbit 1:10000

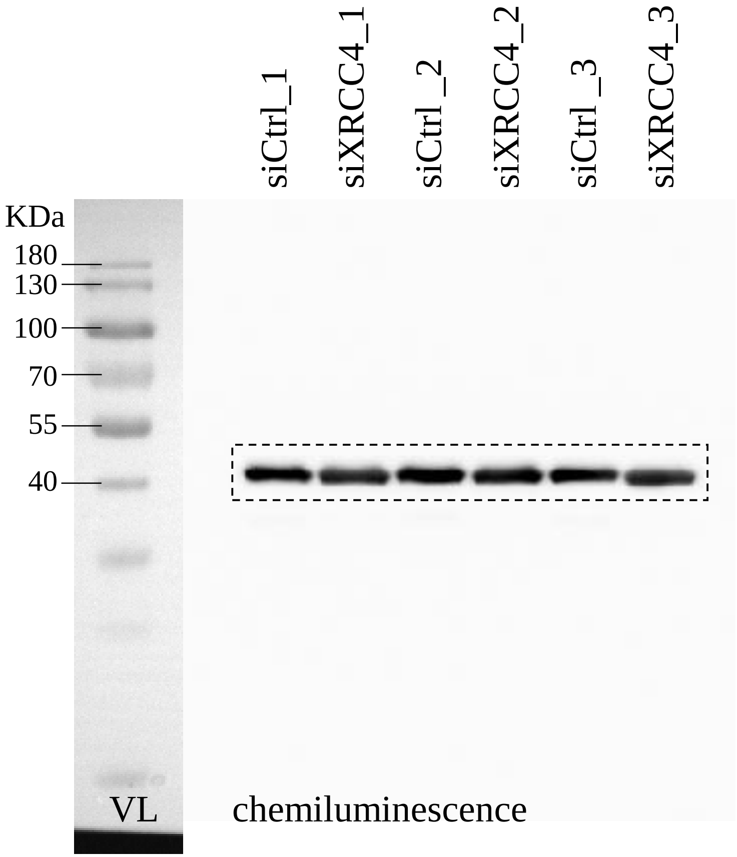

1° anti-**β-actin** 1:10000

2° anti-mouse 1:10000

Part of the image used for the densitometry in the Figure 1.

protein concentration (ug/ul)
siCtrl_1: 5.33, siCtrl _2: 6.19, siCtrl _3: 6.34;
siXRCC4_1: 5.40, siXRCC4_2: 5.93 siXRCC4_3: 6.85.
25ug of cell total protein lysate/lane

Figure S3: Original Western blot images corresponding to Figure 1.

TIG-1

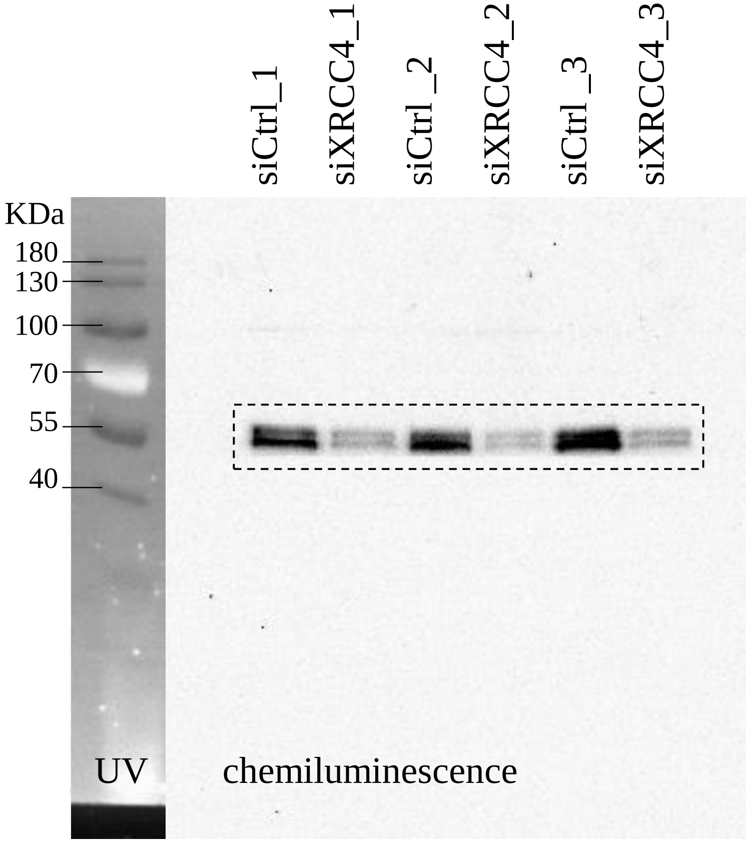

1° anti-**XRCC4** 1:2000

2° anti-mouse 1:10000

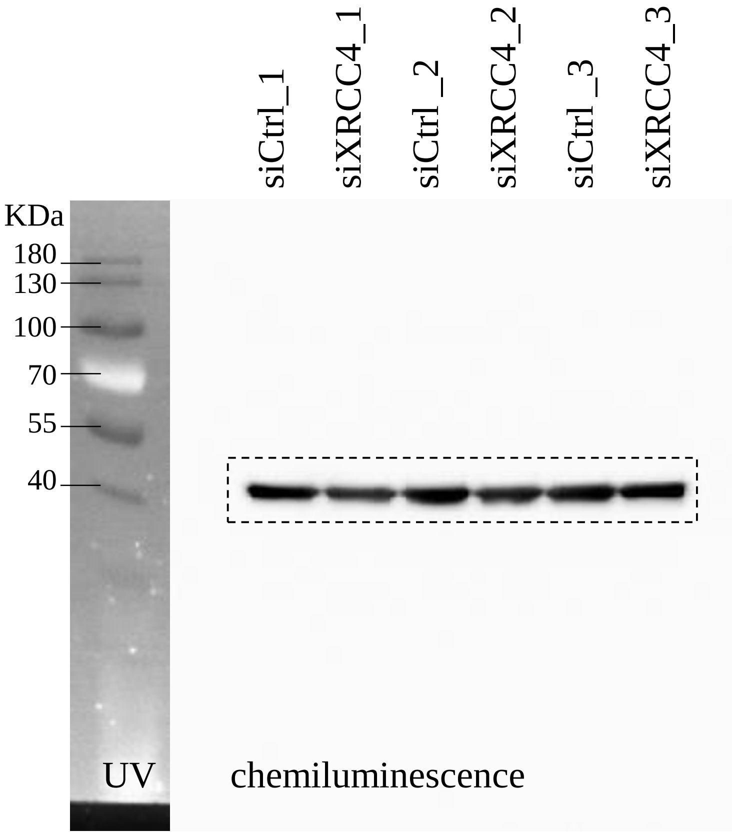

1° anti-**β-actin** 1:10000

2° anti-mouse 1:10000

Part of the image used for the densitometry in the Figure 1.

protein concentration (ug/ul)
siCtrl_1: 4.16, siCtrl _2: 4.04, siCtrl _3: 4.05;
siXRCC4_1: 4.18, siXRCC4_2: 5.07 siXRCC4_3: 4.32.
25ug of cell total protein lysate/lane

Figure S4: Original Western blot images corresponding to Figure 1.

TIG-1

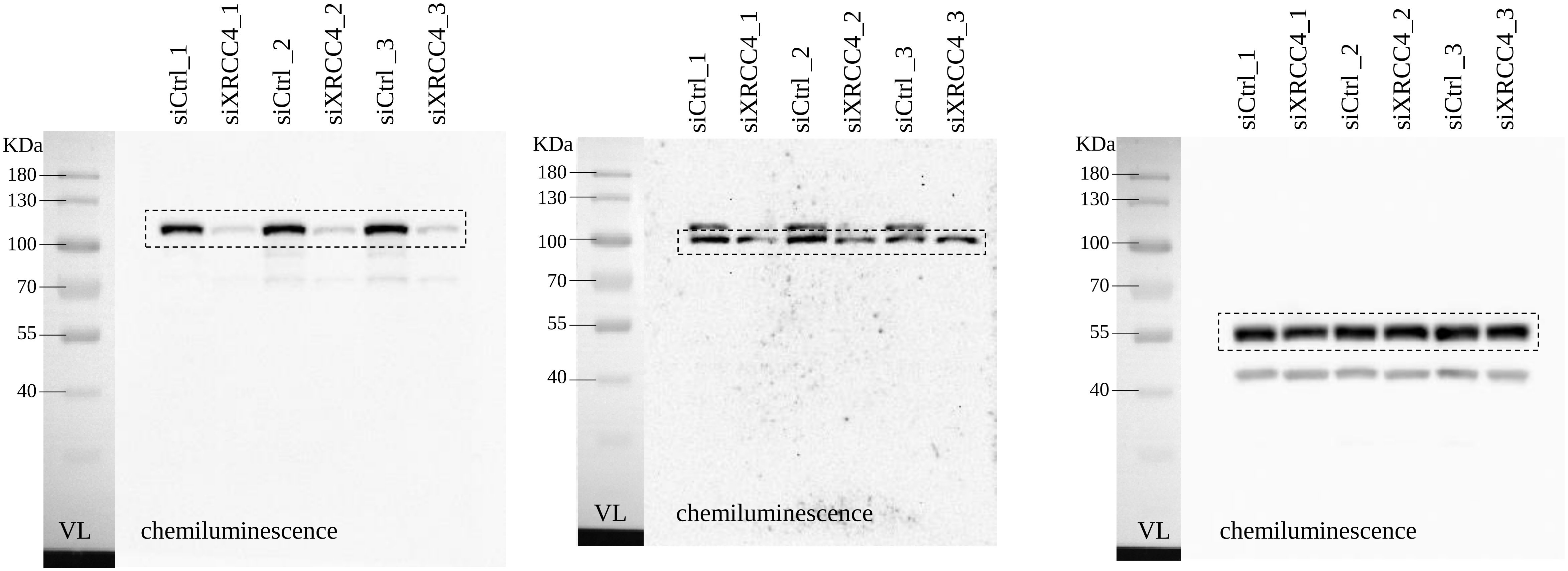

1° anti-**LIG4** 1:1000
2° anti-rabbit 1:10000

1° anti-**LIG3** 1:2000
2° anti-mouse 1:10000

1° anti-**α-tubulin** 1:5000
2° anti-mouse 1:10000

Part of the image used for the densitometry in the Figure 1.

protein concentration (ug/ul)
siCtrl_1: 5.33, siCtrl _2: 6.19, siCtrl _3: 6.34;
siXRCC4_1: 5.40, siXRCC4_2: 5.93 siXRCC4_3: 6.85.
25ug of cell total protein lysate/lane

Figure S5: Original Western blot images corresponding to Figure 1.

NBE1

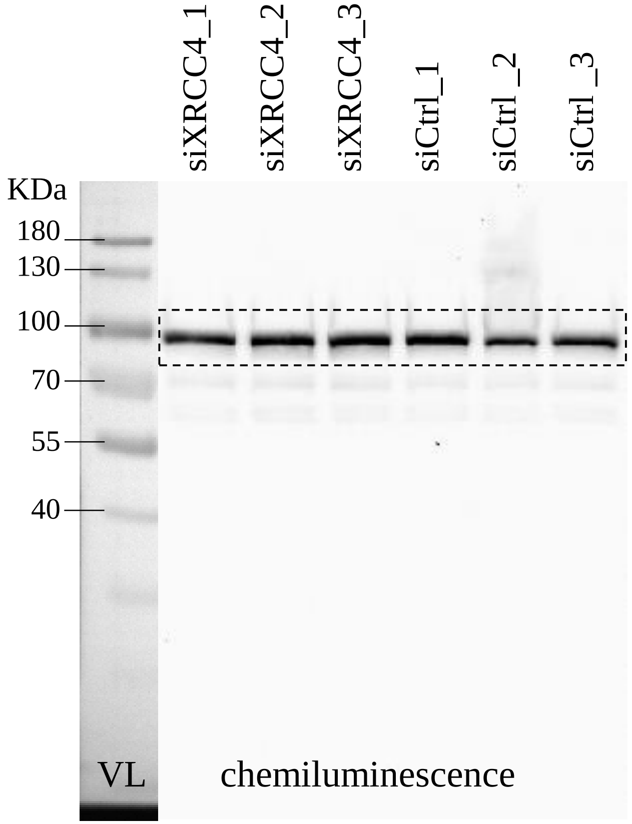

1° anti-**XRCC1** 1:2500

2° anti-mouse 1:10000

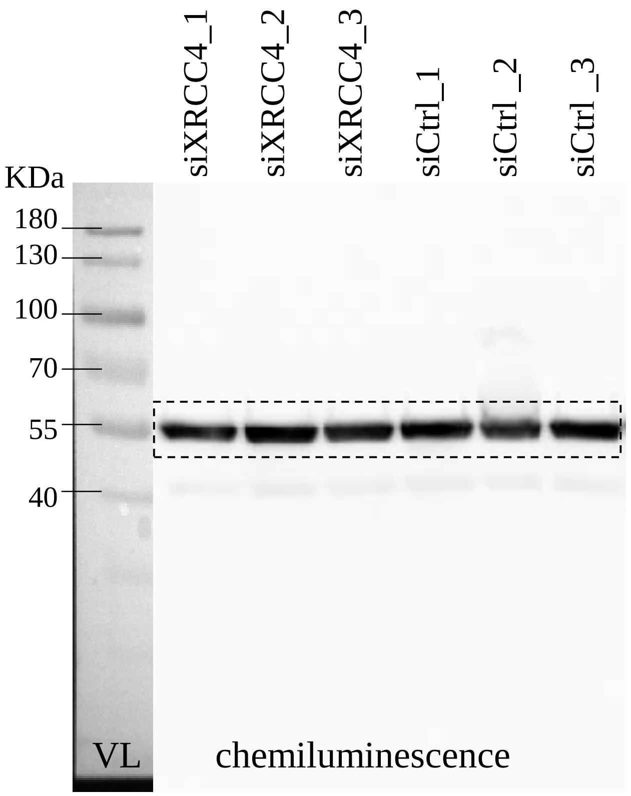

1° anti-**α-tubulin** 1:5000

2° anti-mouse 1:10000

Part of the image used for the densitometry in the Figure 2.

protein concentration (ug/ul)
siCtrl_1: 3.51, siCtrl _2: 3.88, siCtrl _3: 4.26;
siXRCC4_1: 4.13, siXRCC4_2: 4.35 siXRCC4_3: 4.06.
30ug of cell total protein lysate/lane

Figure S6: Original Western blot images corresponding to Figure 2.

NBE1

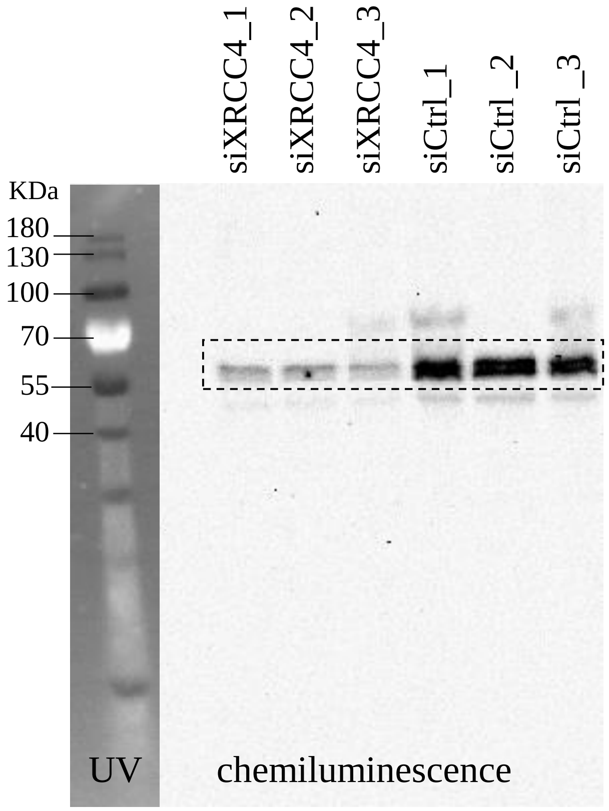

1° anti-**XRCC4** 1:2000

2° anti-mouse 1:10000

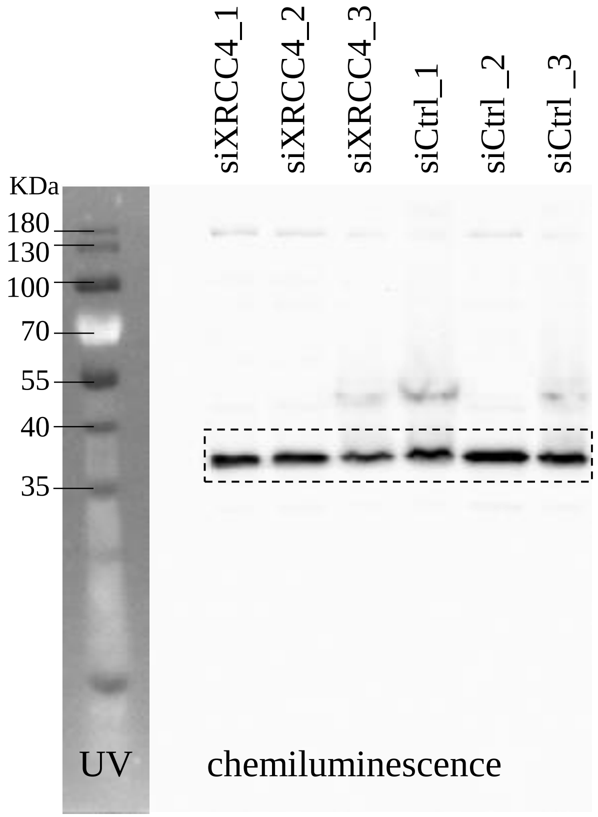

1° anti-**APE1** 1:2000

2° anti-rabbit 1:10000

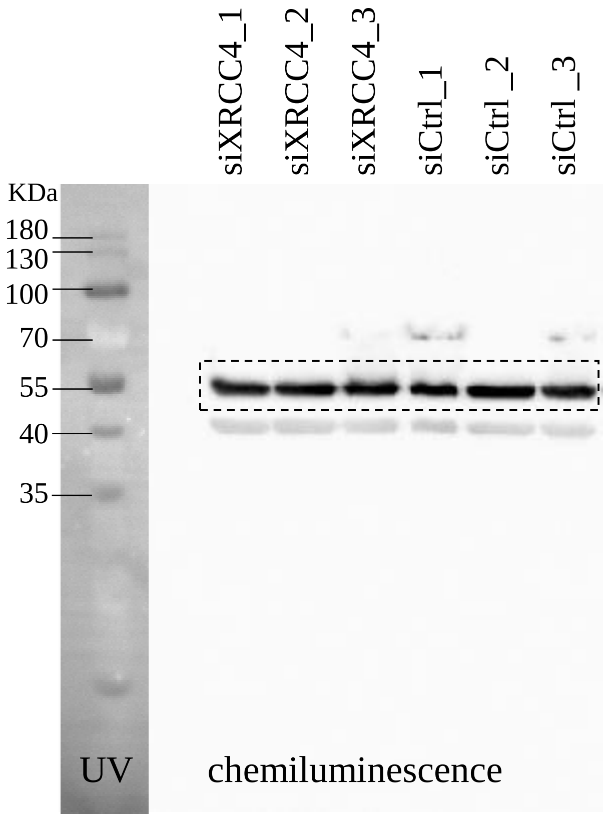

1° anti-**α-tubulin** 1:5000

2° anti-mouse 1:10000

[dashed box] Part of the image used for the densitometry in the Figure 2.

protein concentration (ug/ul)
siCtrl_1: 3.51, siCtrl _2: 3.88, siCtrl _3: 4.26;
siXRCC4_1: 4.13, siXRCC4_2: 4.35 siXRCC4_3: 4.06.
25ug of cell total protein lysate/lane

Figure S7: Original Western blot images corresponding to Figure 2.

NBE1

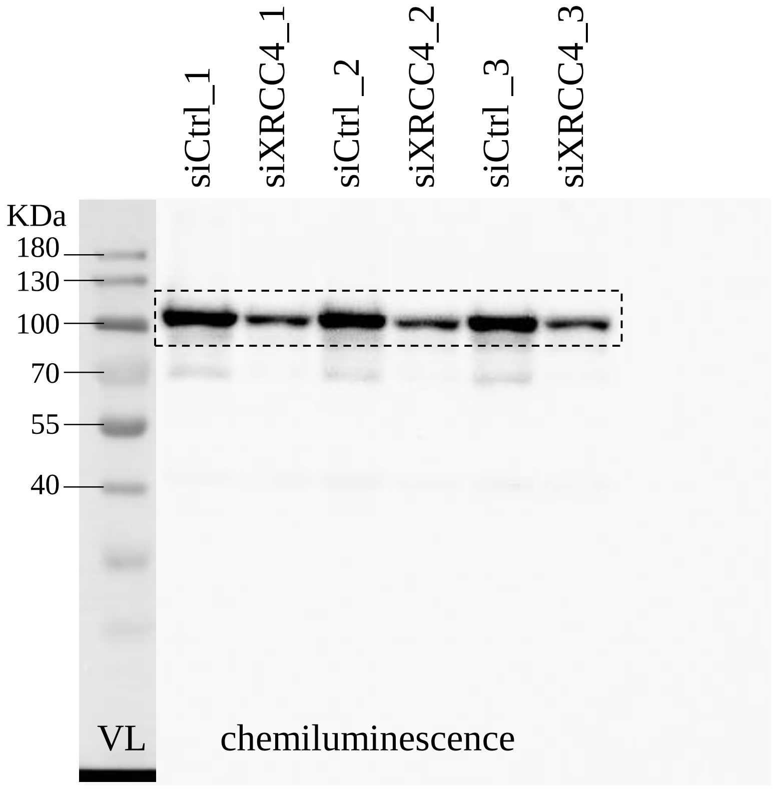

1° anti-**LIG4** 1:1000

2° anti-rabbit 1:10000

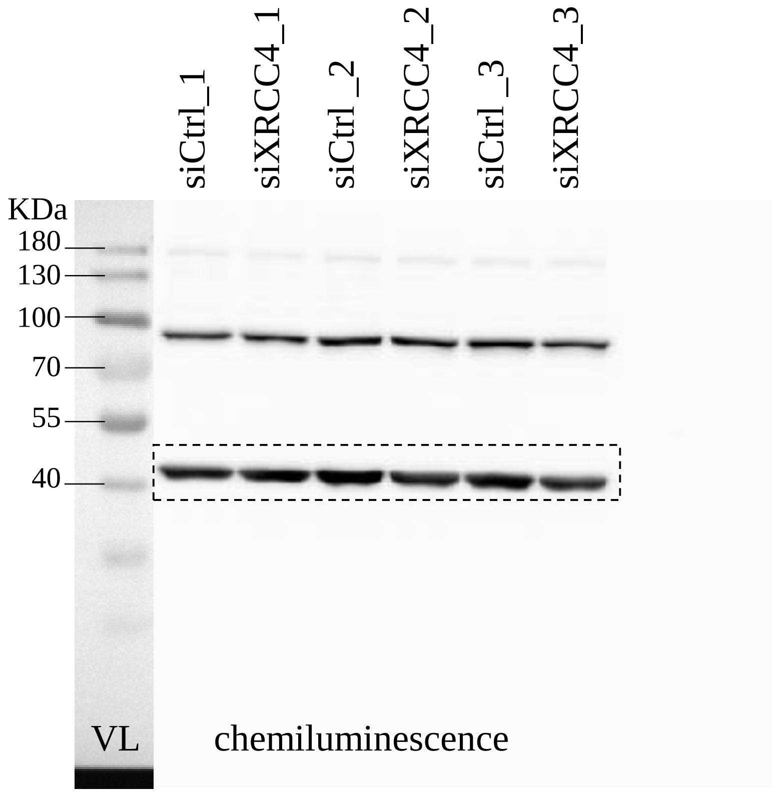

1° anti-**β-actin** 1:10000

2° anti-mouse 1:10000

Part of the image used for the densitometry in the Figure 2.

protein concentration (ug/ul)
siCtrl_1: 3.51, siCtrl _2: 3.88, siCtrl _3: 4.26;
siXRCC4_1: 4.13, siXRCC4_2: 4.35 siXRCC4_3: 4.06.
25ug of cell total protein lysate/lane

Figure S8: Original Western blot images corresponding to Figure 2.

NBE1

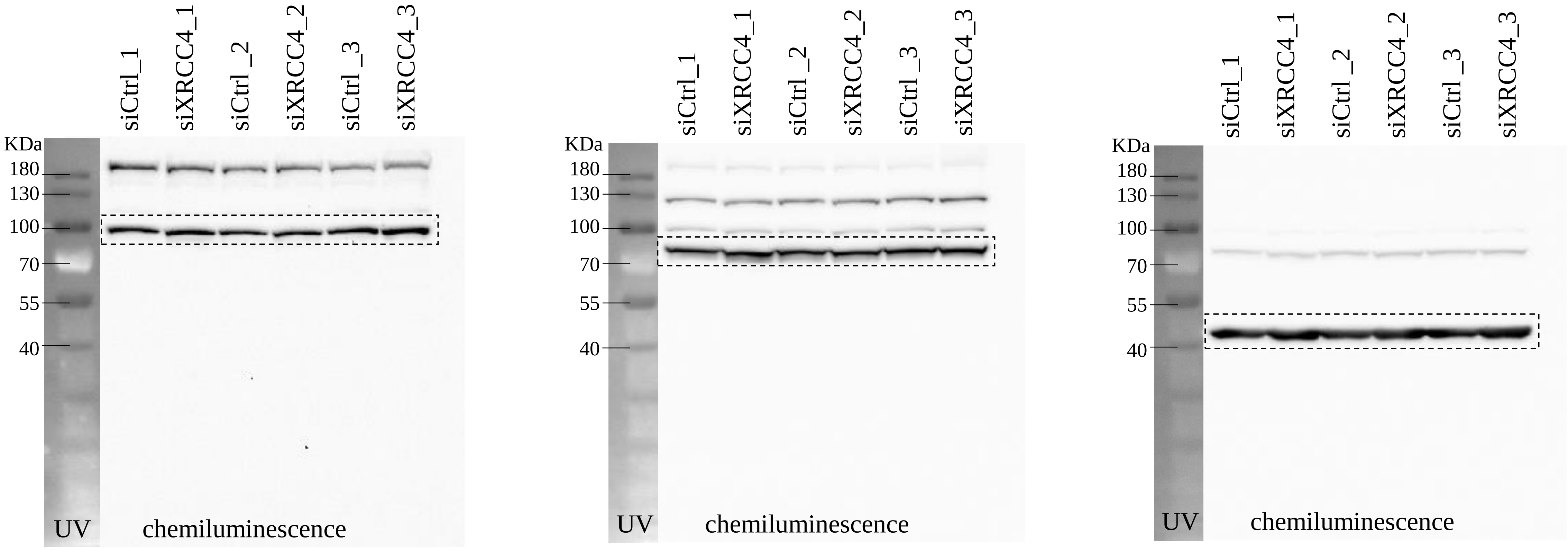

1° anti-**Ku80** 1:1000

2° anti-mouse 1:10000

1° anti-**Ku70** 1:1000

2° anti-mouse 1:10000

1° anti-**β-actin** 1:10000

2° anti-mouse 1:10000

Part of the image used for the densitometry in the Figure 2.

Figure S9: Original Western blot images corresponding to Figure 2.

protein concentration (ug/ul)
siCtrl_1: 8.23, siCtrl _2: 8.53, siCtrl _3: 8.09;
siXRCC4_1: 6.76, siXRCC4_2: 7.10 siXRCC4_3: 6.47.
25ug of cell total protein lysate/lane

NBE1

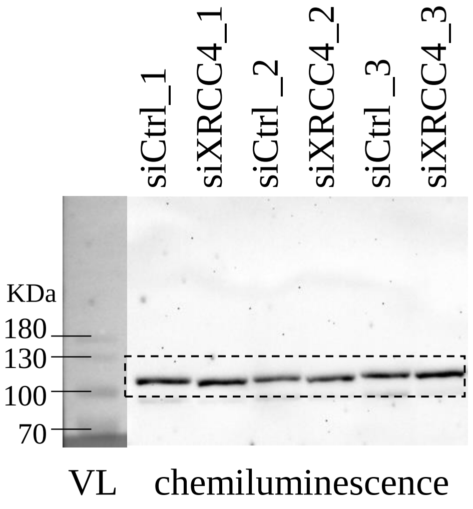

1° anti-**LIG3** 1:2000

2° anti-rabbit 1:10000

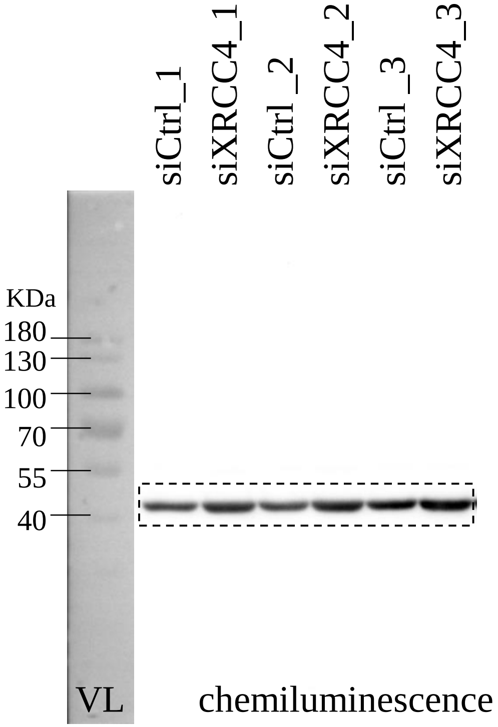

1° anti-**β-actin** 1:10000

2° anti-mouse 1:10000

Part of the image used for the densitometry in the Figure 2.

protein concentration (ug/ul)
siCtrl_1: 8.23, siCtrl _2: 8.53, siCtrl _3: 8.09;
siXRCC4_1: 6.76, siXRCC4_2: 7.10 siXRCC4_3: 6.47.
25ug of cell total protein lysate/lane

Figure S10: Original Western blot images corresponding to Figure 2.

NBE1

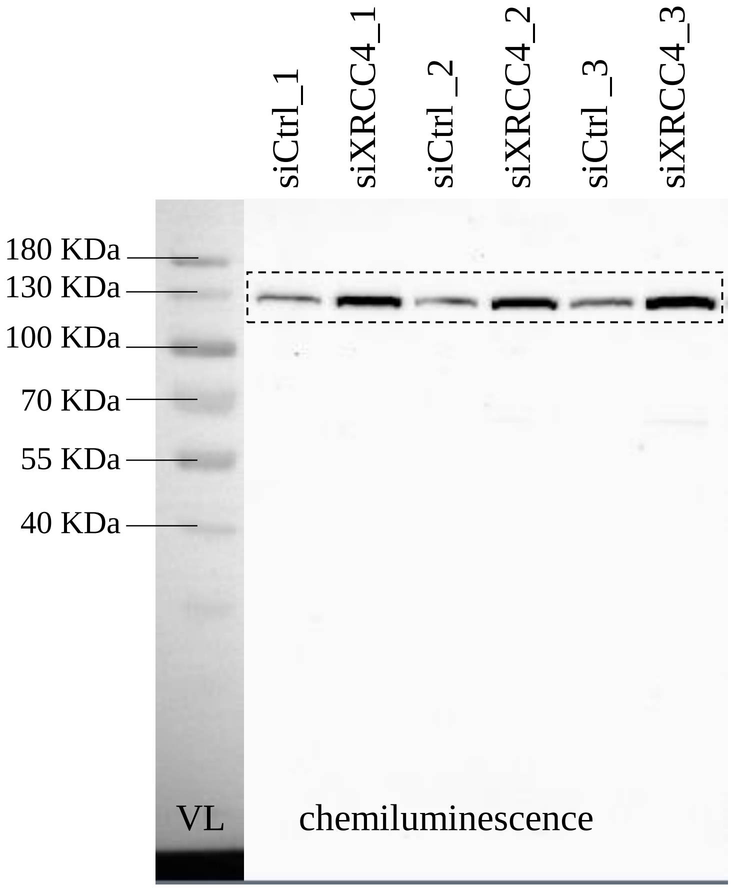

1° anti-**PARP1** 1:500

2° anti-rabbit 1:10000

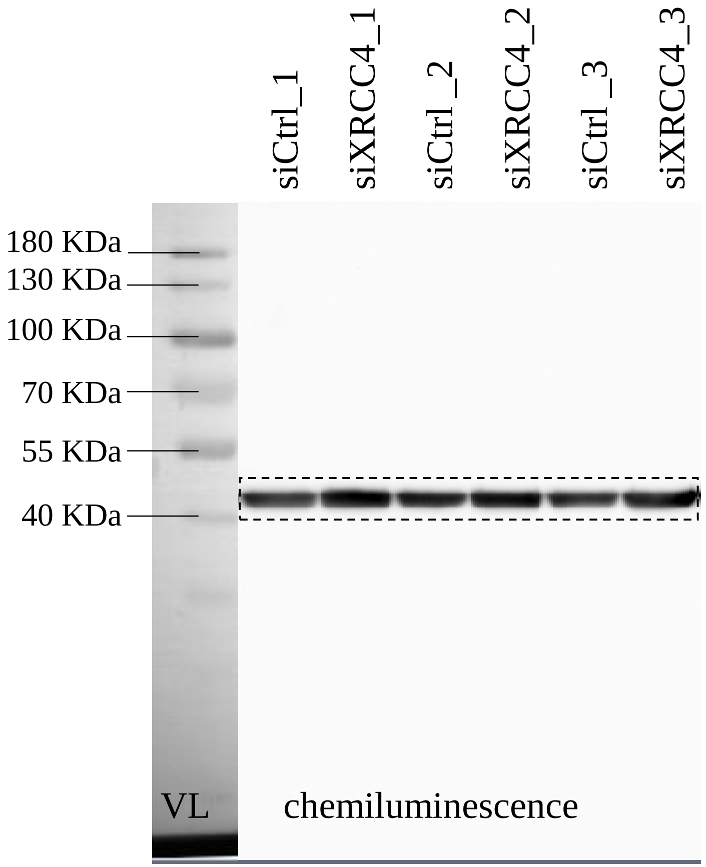

1° anti-**β-actin** 1:10000

2° anti-mouse 1:10000

Part of the image used for the densitometry in the Figure 4 for NBE1 cells.

protein concentration (ug/ul)
siCtrl_1: 8.23, siCtrl _2: 8.53, siCtrl _3: 8.09;
siXRCC4_1: 6.76, siXRCC4_2: 7.10 siXRCC4_3: 6.47.
25ug of cell total protein lysate/lane

Figure S11: Original Western blot images corresponding to Figure 4 for NBE1 cells.

TIG-1

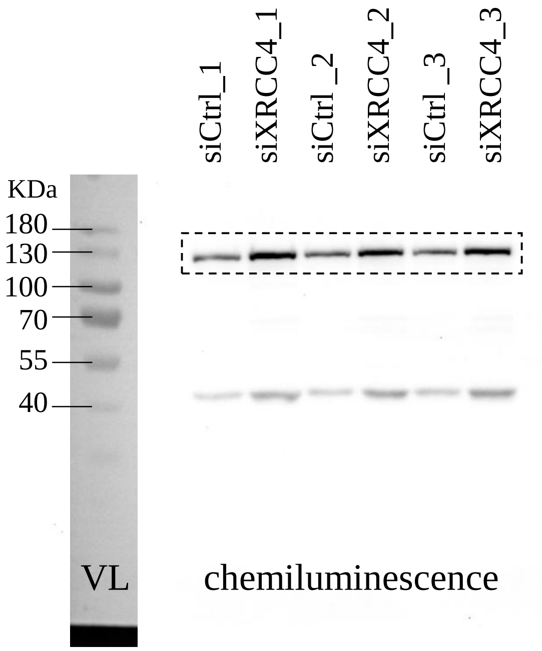

1° anti-**PARP1** 1:500

2° anti-rabbit 1:10000

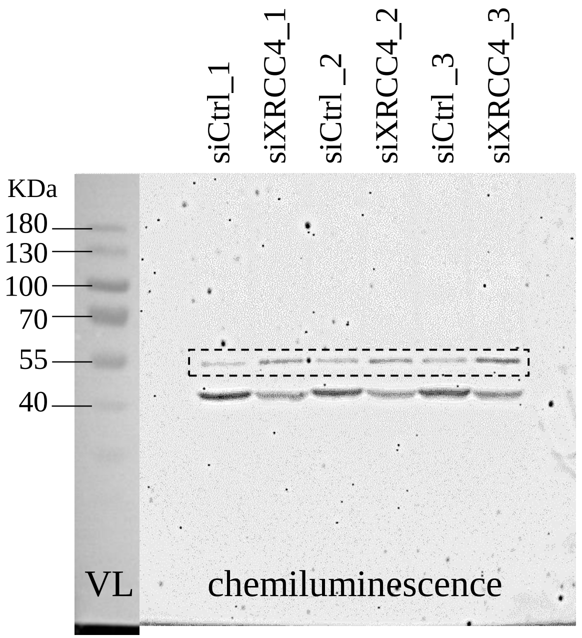

1° anti-**p53** 1:500

2° anti-mouse 1:10000

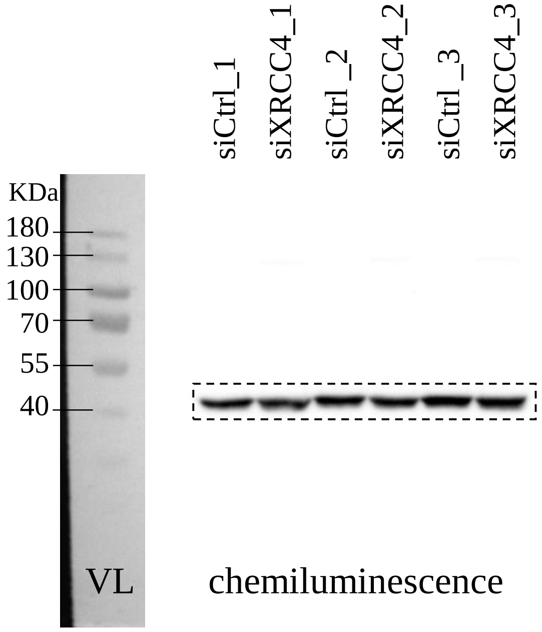

1° anti-**β-actin** 1:10000

2° anti-mouse 1:10000

Part of the image used for the densitometry in the Figure 4 and Figure 5 for TIG-1 cells.

Figure S12: Original Western blot images corresponding to Figure 1 and Figure 5 for TIG-1 cells.

protein concentration (ug/ul)
siCtrl_1: 5.33, siCtrl _2: 6.19, siCtrl _3: 6.34;
siXRCC4_1: 5.40, siXRCC4_2: 5.93 siXRCC4_3: 6.85.
25ug of cell total protein lysate/lane

TIG-1

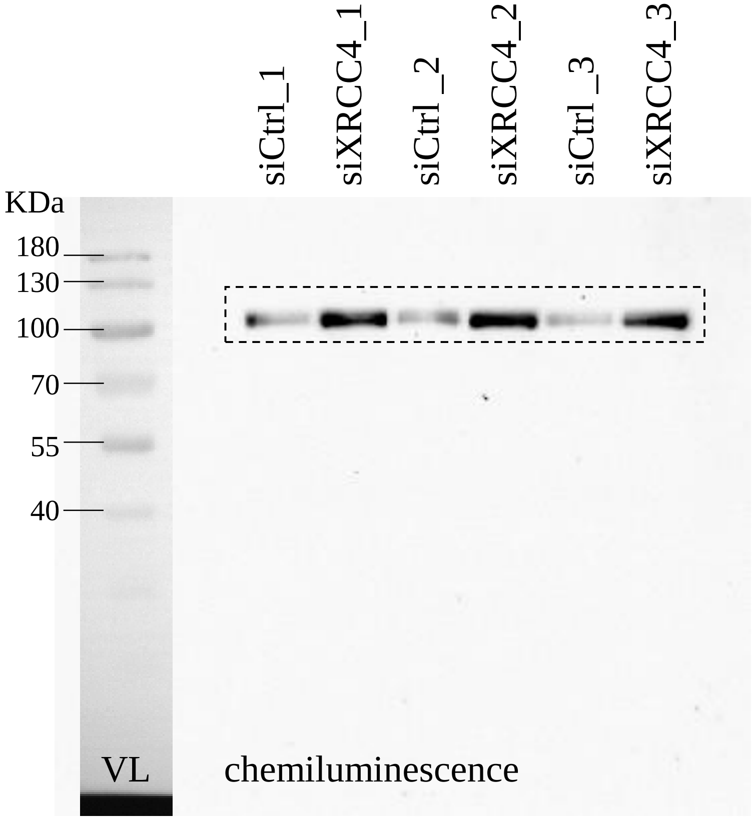

1° anti-**Sp1** 1:1000

2° anti-rabbit 1:10000

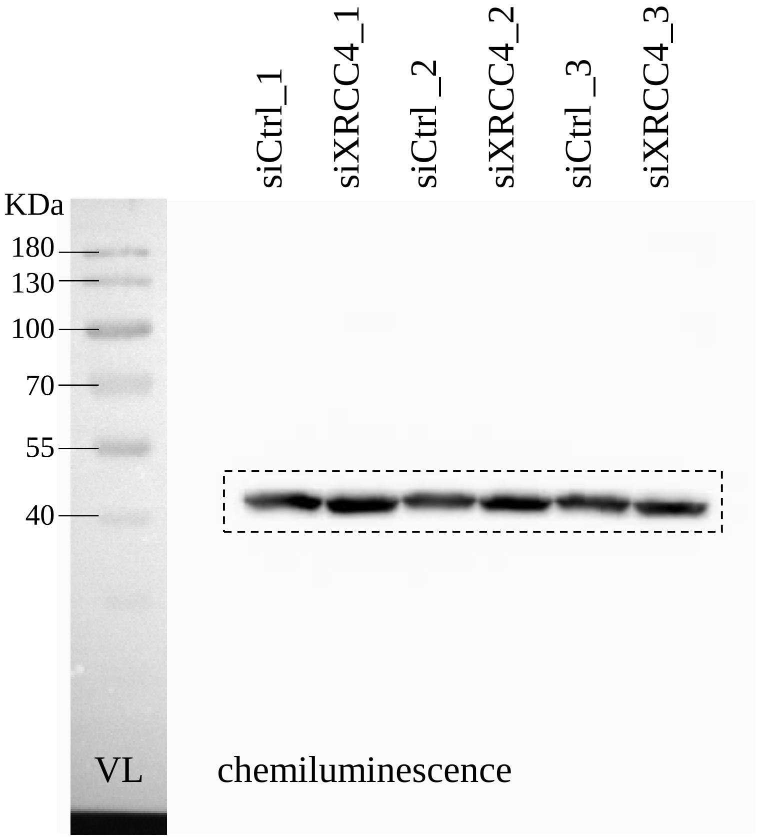

1° anti-**β-actin** 1:10000

2° anti-mouse 1:10000

Part of the image used for the densitometry in the Figure 5 for TIG-1 cells.

protein concentration (ug/ul)
siCtrl_1: 5.33, siCtrl _2: 6.19, siCtrl _3: 6.34;
siXRCC4_1: 5.40, siXRCC4_2: 5.93 siXRCC4_3: 6.85.
25ug of cell total protein lysate/lane

Figure S13: Original Western blot images corresponding to Figure 5 for TIG-1 cells.

NBE1

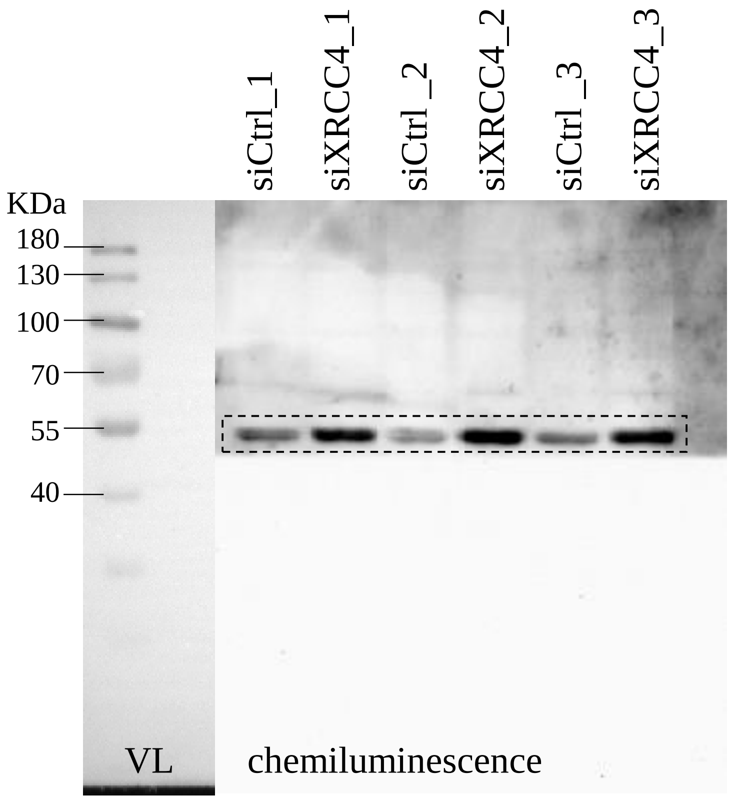

1° anti-**p53** 1:500

2° anti-mouse 1:10000

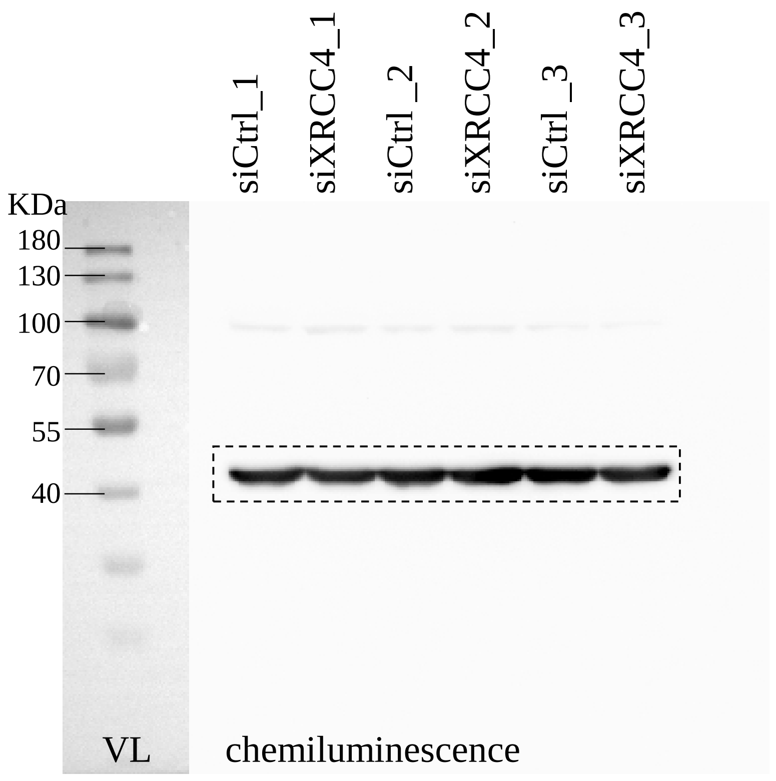

1° anti-**β-actin** 1:10000

2° anti-mouse 1:10000

Part of the image used for the densitometry in the Figure 5 for NBE1 cells.

protein concentration (ug/ul)
siCtrl_1: 3.51, siCtrl _2: 3.88, siCtrl _3: 4.26;
siXRCC4_1: 4.13, siXRCC4_2: 4.35 siXRCC4_3: 4.06.
30ug of cell total protein lysate/lane

Figure S14: Original Western blot images corresponding to Figure 5 for NBE1 cells.

NBE1

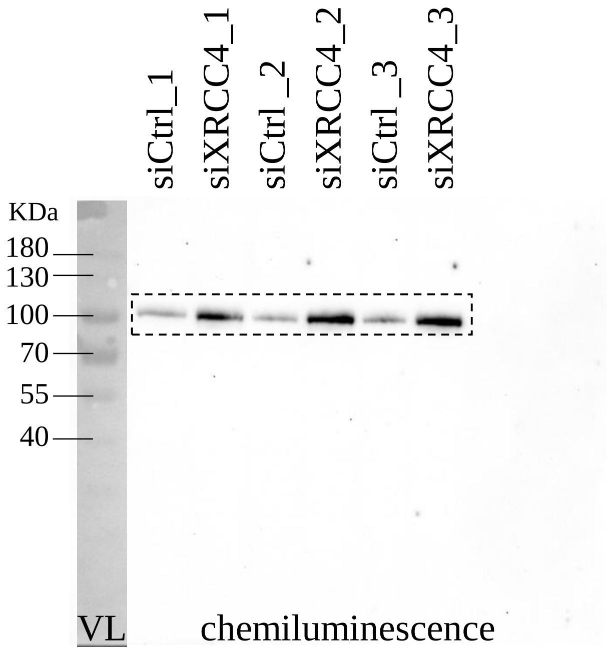

1° anti-**Sp1** 1:1000

2° anti-rabbit 1:10000

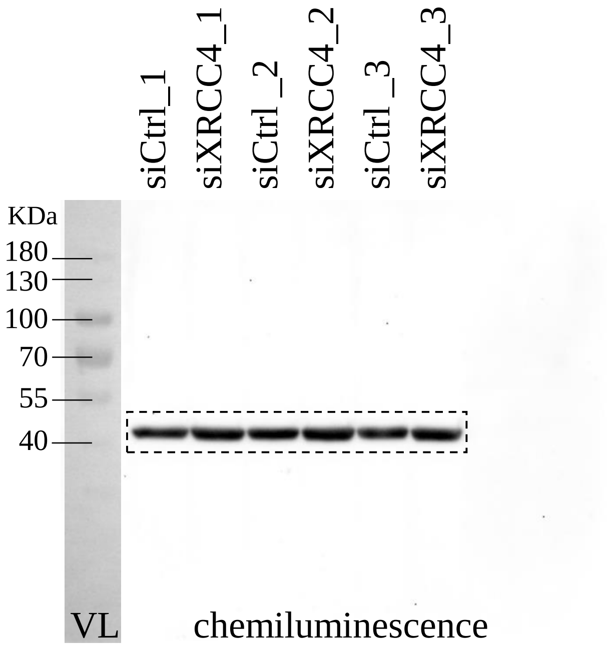

1° anti-**β-actin** 1:10000

2° anti-mouse 1:10000

Part of the image used for the densitometry in the Figure 5 for NBE1 cells.

protein concentration (ug/ul)
siCtrl_1: 8.23, siCtrl _2: 8.53, siCtrl _3: 8.09;
siXRCC4_1: 6.76, siXRCC4_2: 7.10 siXRCC4_3: 6.47.
25ug of cell total protein lysate/lane

Figure S15: Original Western blot images corresponding to Figure 5 for NBE1 cells.

TIG-1

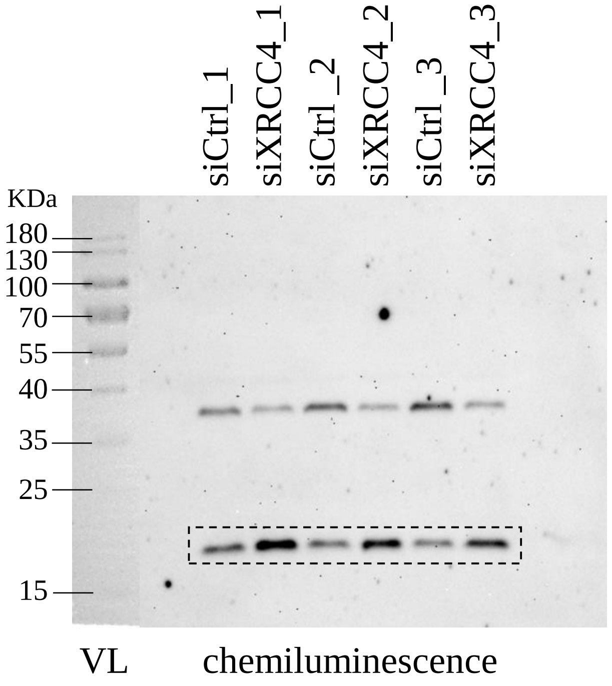

1° anti-**p21** 1:1000

2° anti-rabbit 1:10000

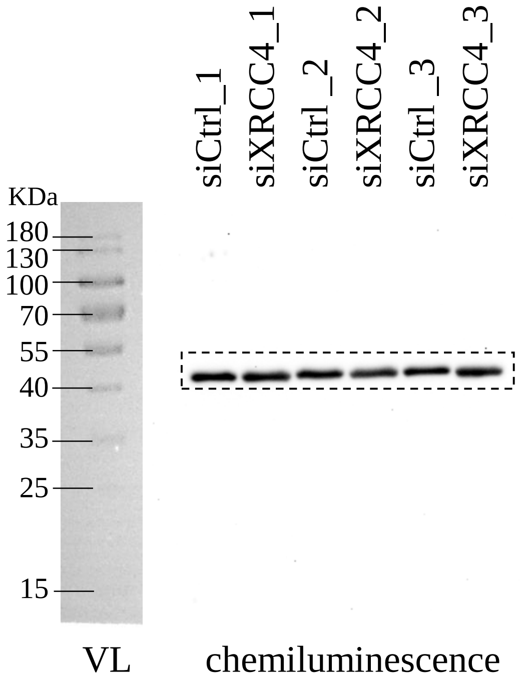

1° anti-**β-actin** 1:10000

2° anti-mouse 1:10000

Part of the image used for the densitometry in the Figure 7 for TIG-1 cells.

protein concentration (ug/ul)
siCtrl_1: 5.33, siCtrl _2: 6.19, siCtrl _3: 6.34;
siXRCC4_1: 5.40, siXRCC4_2: 5.93 siXRCC4_3: 6.85.
25ug of cell total protein lysate/lane

Figure S16: Original Western blot images corresponding to Figure 7 for TIG-1 cells.

NBE1

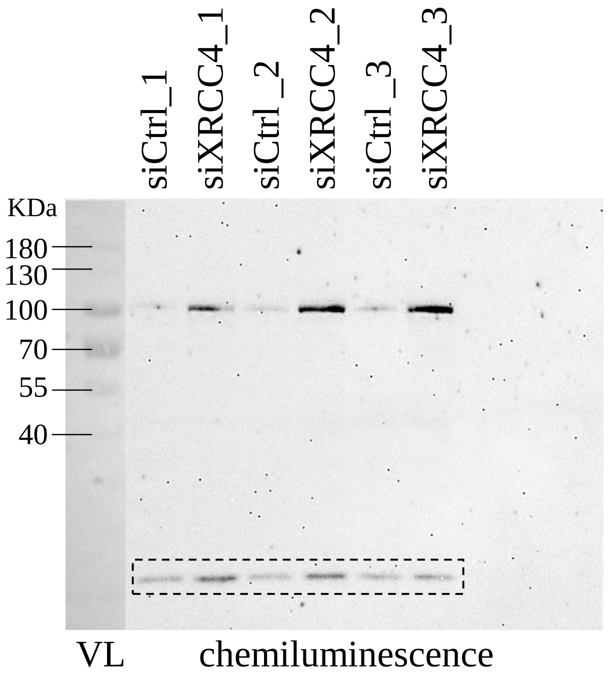

1° anti-**p21** 1:1000

2° anti-rabbit 1:10000

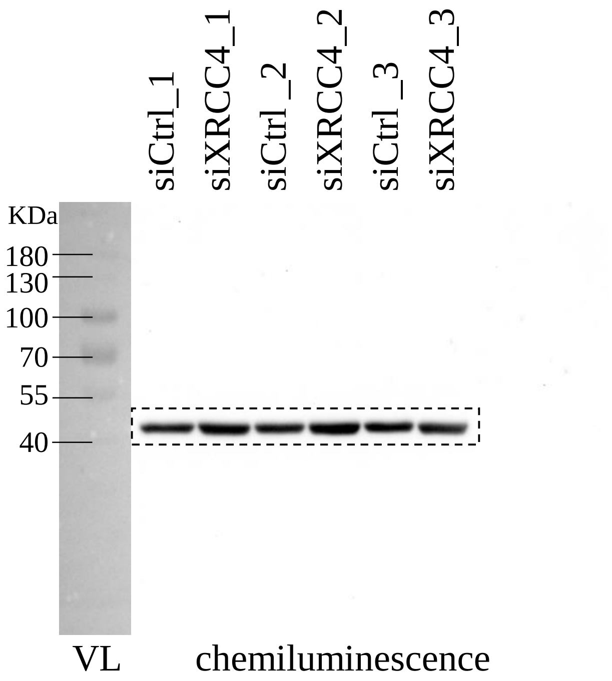

1° anti-**β-actin** 1:10000

2° anti-mouse 1:10000

Part of the image used for the densitometry in the Figure 7 for NBE1 cells.

protein concentration (ug/ul)
siCtrl_1: 8.23, siCtrl _2: 8.53, siCtrl _3: 8.09;
siXRCC4_1: 6.76, siXRCC4_2: 7.10 siXRCC4_3: 6.47.
25ug of cell total protein lysate/lane

Figure S17: Original Western blot images corresponding to Figure 7 for NBE1 cells.
